# Linear growth trajectories in Zimbabwean infants^1^,^2^

**DOI:** 10.3945/ajcn.116.133538

**Published:** 2016-11-02

**Authors:** Ethan K Gough, Erica EM Moodie, Andrew J Prendergast, Robert Ntozini, Lawrence H Moulton, Jean H Humphrey, Amee R Manges

**Affiliations:** 3 Department of Epidemiology, Biostatistics and Occupational Health, McGill University, Montreal, Canada;; 4 Zvitambo Institute for Maternal Child Health Research, Harare, Zimbabwe;; 5 Blizard Institute, Queen Mary University of London, London, United Kingdom;; 6 Department of International Health, Johns Hopkins Bloomberg School of Public Health, Baltimore, MD; and; 7 School of Population and Public Health, University of British Columbia, Vancouver, Canada

**Keywords:** children, infants, longitudinal, malnutrition, prenatal, stunting

## Abstract

**Background:** Undernutrition in early life underlies 45% of child deaths globally. Stunting malnutrition (suboptimal linear growth) also has long-term negative effects on childhood development. Linear growth deficits accrue in the first 1000 d of life. Understanding the patterns and timing of linear growth faltering or recovery during this period is critical to inform interventions to improve infant nutritional status.

**Objective:** We aimed to identify the pattern and determinants of linear growth trajectories from birth through 24 mo of age in a cohort of Zimbabwean infants.

**Design:** We performed a secondary analysis of longitudinal data from a subset of 3338 HIV-unexposed infants in the Zimbabwe Vitamin A for Mothers and Babies trial. We used *k*-means clustering for longitudinal data to identify linear growth trajectories and multinomial logistic regression to identify covariates that were associated with each trajectory group.

**Results:** For the entire population, the mean length-for-age *z* score declined from −0.6 to −1.4 between birth and 24 mo of age. Within the population, 4 growth patterns were identified that were each characterized by worsening linear growth restriction but varied in the timing and severity of growth declines. In our multivariable model, 1-U increments in maternal height and education and infant birth weight and length were associated with greater relative odds of membership in the least–growth restricted groups (A and B) and reduced odds of membership in the more–growth restricted groups (C and D). Male infant sex was associated with reduced odds of membership in groups A and B but with increased odds of membership in groups C and D.

**Conclusion:** In this population, all children were experiencing growth restriction but differences in magnitude were influenced by maternal height and education and infant sex, birth weight, and birth length, which suggest that key determinants of linear growth may already be established by the time of birth. This trial was registered at clinicaltrials.gov as NCT00198718.

## INTRODUCTION

Undernutrition underlies 45% of mortality in children aged <5 y worldwide, which results in 3.1 million deaths annually (1). Linear growth faltering in children (growth in height or length) is viewed as an indicator of long-term nutritional status (2). Children whose linear growth [expressed as a length-for-age *z* score (LAZ)^9^] is >2 SDs below the WHO standard population median are termed stunted (2). In addition to its short-term effects on morbidity and mortality, stunting also contributes to poor motor development, cognition, educational achievement, and economic attainment over the life course (1, 3, 4). Despite a modest decrease in the global prevalence of stunting since 1990, an estimated 165 million children <5 y old were stunted in 2011 (1), representing approximately one-third of children in this age group in developing countries.

An estimated 20% of linear growth faltering occurs in utero (5). Although there is a wide variation between countries, 11–16% of newborns in developing countries are born with low birth weight (LBW) (i.e., birth weight <2500 g), and 27% of newborns are small for gestational age (SGA), which is defined as being <10th percentile in weight for infant gestational age and sex (6, 7). Infants who are born small show increased risk of stunting during infancy (5, 8–16). Analyses of cross-sectional data from 54 countries have shown that the mean LAZ in Africa and Asia is below the WHO standard median at birth and progressively declines through 24 mo of age with little to no recovery thereafter (17). However, growth trajectories vary across populations (18–21). In addition, there is considerable variability in growth restriction or recovery in individual children during the first 2–3 y after birth, which suggests that meaningful departures from population mean growth do occur (22–24). The aggregation of data within populations and across countries may obscure differences in growth patterns between individuals.

Interventions to prevent stunting need to be targeted early in the life course during the critical window from conception through a child’s second birthday (the so-called first 1000 d; www.thousanddays.org) (25, 26). However, an understanding of the temporal patterns of linear growth in early life, the timing of growth faltering or recovery, and the factors that determine which growth pattern an infant experiences are critical to inform the precise nature and timing of interventions to promote healthy growth. In this analysis, we aimed to characterize the linear growth trajectories of HIV-unexposed Zimbabwean infants from birth through 2 y of age by clustering infants with similar longitudinal growth patterns. We identified sociodemographic and epidemiologic factors that are associated with membership in each trajectory group with the aim of better understanding the patterns and determinants of growth in the first 1000 d of life.

## METHODS

### Study population

We used data from the Zimbabwe Vitamin A for Mothers and Babies (clinicaltrials.gov; NCT00198718) study, which is a randomized, placebo-controlled trial of peripartum vitamin A supplementation that has been previously described (27, 28). In brief, 14,110 mother-infant pairs were enrolled at 14 maternity clinics and hospitals in Harare, Zimbabwe, between 1997 and 2001. Study participants were recruited ≤96 h after delivery and were followed up when infants were 6 wk and 3 mo old and every 3 mo afterward until age 12–24 mo in a dedicated study clinic. Data on maternal education, sociodemographic variables, anthropometric measures, and paternal education were collected at baseline. Infant sociodemographic information and history of sick clinic visits and hospitalizations were collected with the use of questionnaires and through the transcription of data from health-facility records. Infant weight and length were measured at each visit with the use of an electronic scale (Seca Model 727; seca) and length board (ShorrBoard; Weigh and Measure LLC), respectively, according to the methods described by Gibson (29).

**FIGURE 1:**
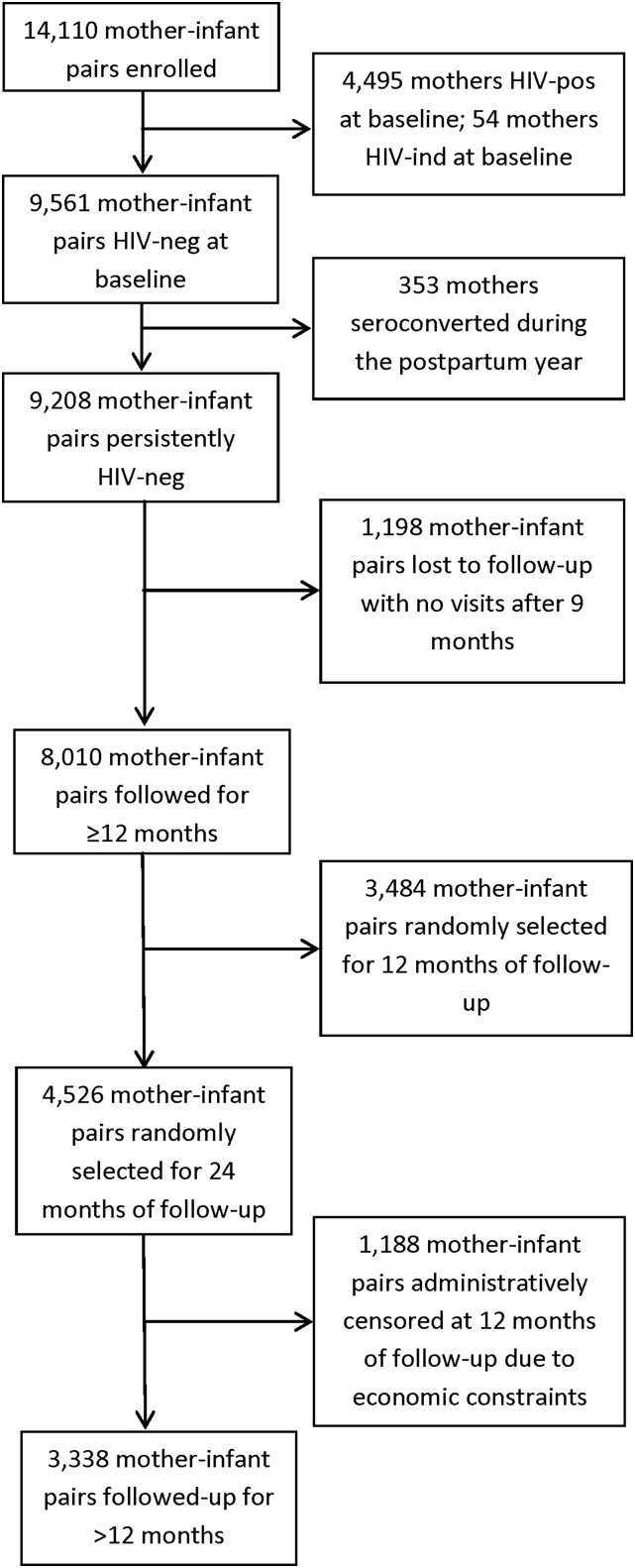
Patient flowchart. HIV-ind, HIV indeterminate; HIV-neg, HIV negative; HIV-pos, HIV positive.

A total of 9208 infants were HIV unexposed, which meant that they were born to mothers who tested HIV negative at baseline and remained HIV negative throughout the follow-up. The trial planned to follow a random subset of one-half of these HIV-unexposed infants through 24 mo of infant age. However, because of economic constraints in June 2000, follow-up was terminated at age 12 mo or, for those already beyond that visit, at the next 3-mo follow-up (administrative censorship). Thus, only 3338 HIV-negative mother-infant pairs were followed for >12 mo (**Figure 1**). We restricted our analyses to this subset of participants and performed a prospective cohort study to investigate linear growth beyond infancy in an HIV-unexposed infant population.

The Medical Research Council of Zimbabwe, Medicines Control Authority of Zimbabwe, Johns Hopkins Bloomberg School of Public Health Committee on Human Research, and Montreal General Hospital Ethics Committee approved the Zimbabwe Vitamin A for Mothers and Babies trial protocol. Ethical review and approval were obtained annually from the Medical Research Council of Zimbabwe for ongoing use of trial data, and ethical approval for these analyses was obtained from the McGill University Ethical Review Board.

### Definition of variables

Growth was expressed as the LAZ or weight-for-length *z* score with the use of the WHO growth standard (2) with WHO Anthro version 3.0.1 software (http://www.who.int/childgrowth/en; WHO). Gestational age was estimated with the use of the method described by Capurro et al. (30). Breastfeeding between birth and 3 mo of age was categorized as exclusive, predominant, or mixed as previously defined (31). Clinic visits for the treatment of illness and hospitalizations were defined as the total number of sick clinic visits or hospital admissions during the period since the previous scheduled follow-up visit.

### Clustering of longitudinal length-for-age growth trajectories

We used *k*-means clustering for longitudinal data (KML) (32) to identify groups of infants who were most similar to each other in the level as well as the shape of their LAZ growth curves over time and to separate infants with different growth patterns. KML can identify distinct growth groups even if they overlap and if their trajectories cross and has fewer assumptions than other methods, and thus, it is less susceptible to biases if assumptions do not hold. We used the Euclidean distance adjusted for temporal correlations to measure the growth-curve similarity accounting for both similarities in LAZ values and temporal behavior (33). Because we had no a priori knowledge of the number of distinct trajectory groups in this cohort, we searched for a minimum of 2 groups to a possible maximum of 9 groups. For each possible number of groups, we reran the KML algorithm 1000 times to search for the most-optimal cluster result. To choose the optimal cluster result, the Caliński-Harabasz index was used as the measure of cluster quality (34). An increase in this index indicates greater separation between groups and greater similarity within groups, thereby indicating better clustering. The optimal number of clusters and best cluster result from the 1000 runs were chosen as the ones that produced the largest inflection in the Calinksi-Harabasz index.

### Multiple imputation of unobserved longitudinal data

Because of the administrative censorship as well as losses to follow-up and incomplete anthropometric measures, there was a notable amount of unobserved length data overall (**Table 1**). Of 3338 infants included in the analysis, ∼30% of the children were missing a length measure at each time point, and 1834 infants (55%) were censored before 24 mo of age. Because *k*-means clustering requires complete data, we applied a data-imputation strategy with the use of multiple imputation by chained equations (35) to generate complete data sets. We used predictive mean matching (36) with all available baseline variables (presented in Table 1) included in the imputation model together with the LAZ at each visit, weight-for-length *z* score at each visit, breastfeeding pattern from birth to age 3 mo, infant age, follow-up visit number, and the total number of sick clinic visits and hospitalizations at each follow-up visit. We also included an indicator variable for administrative censorship and the total number of sick clinic visits at each follow-up that were due to acute respiratory infections in the imputation model because these variables were strongly correlated with missingness.

**TABLE 1:** Description of the cohort (*n* = 3338)^1^

		Mean (95% CI)	
	Missing,^2^ %	Observed^3^	Imputed^4^
Vitamin A treatment arm,^5^ %			
Maternal treatment–infant treatment	0.0	25.1 (23.6, 26.5)	—
Maternal treatment–infant placebo	0.0	24.4 (23.0, 25.9)	—
Maternal placebo–infant treatment	0.0	24.9 (23.4, 26.3)	—
Maternal placebo–infant placebo	0.0	25.6 (24.1, 27.1)	—
Maternal age, y	0.2	24.2 (24.0, 24.3)	—
Maternal education, y	0.1	9.7 (9.7, 9.8)	—
Maternal height, cm	30.5	160.1 (159.8, 160.4)	160.1 (159.8, 160.4)
Maternal MUAC, cm	0.6	25.9 (25.8, 26.0)	—
Cesarean delivery, %	1.0	8.5 (7.5, 9.5)	—
Time since last birth,^6^ y	0.9	4.3 (4.2, 4.4)	—
Children, *n*	0.0	2.0 (1.9, 2.0)	—
Parity, %			
Primiparous	0.0	49.7 (48.0, 51.4)	—
Multiparous (2–3)	0.0	37.5 (35.8, 39.1)	—
Multiparous (≥4)	0.0	12.8 (11.7, 14.0)	—
Paternal education, y	2.2	10.6 (10.6, 10.7)	—
Infant sex, M, %	0.1	51.9 (50.2, 53.6)	—
Gestational age, wk	0.8	39.3 (39.3, 39.4)	—
Term and AGA, %	0.8	74.9 (72.8, 75.8)	—
Preterm and AGA, %	0.8	5.3 (4.5, 6.0)	—
Term and SGA, %	0.8	19.4 (18.0, 20.7)	—
Preterm and SGA, %	0.8	1.1 (0.7, 1.4)	—
Birth length, cm	1.1	48.4 (48.3, 48.5)	—
Birth weight, g	0.3	2995 (2980, 3010)	—
Birth weight <2500 g, %	0.3	12.4 (11.3, 13.6)	—
Time until first breastfeeding, h	2.8	2.8 (2.6, 3.0)	—
Breastfeeding,^7^ %			
None	0.0	38.6 (37.0, 40.3)	—
Exclusive	0.0	2.6 (2.1, 3.1)	—
Partial	0.0	15.3 (14.1, 16.5)	—
Mixed	0.0	43.4 (41.8, 45.1)	—
Clinic visits/infant by age in mo,^8^ *n*			
Birth to 6	ND	1.14 (1.10, 1.17)	—
6–12	ND	0.81 (0.78, 0.84)	—
12–18	ND	0.60 (0.57, 0.62)	—
18–24	ND	0.31 (0.29, 0.33)	—
Hospital admissions/infant by age in mo,^8^ *n*			
Birth to 6	ND	0.05 (0.04, 0.05)	—
6–12	ND	0.02 (0.02, 0.03)	—
12–18	ND	0.02 (0.01, 0.02)	—
18–24	ND	0.01 (0.01, 0.02)	—
WLZ, by age in mo			
Birth	8.2	−0.40 (−0.46, −0.35)	—
6	27.4	0.43 (0.36, 0.48)	—
12	45.5	0.08 (0.02, 0.13)	—
18	50.5	−0.01 (−0.06, 0.04)	—
24	65.4	0.02 (−0.05, 0.08)	—
LAZ, by age in mo			
Birth	1.7	−0.62 (−0.66, −0.58)	−0.62 (−0.66, −0.58)
6	27.1	−0.75 (−0.79, −0.70)	−0.73 (−0.77, −0.68)
12	45.5	−1.03 (−1.09, −0.98)	−1.02 (−1.15, −0.88)
18	50.5	−1.33 (−1.39, −1.28)	−1.31 (−1.37, −1.25)
24	65.3	−1.42 (−1.49, −1.36)	−1.38 (−1.69, −1.07)
Stunted,^9^ by age in mo, %			
Birth	1.7	11.8 (10.7, 12.9)	11.7 (10.6, 12.8)
6	27.1	14.0 (12.6, 15.4)	13.6 (12.3, 14.9)
12	45.5	19.0 (17.2, 20.8)	19.1 (16.0, 22.2)
18	50.5	26.8 (24.7, 28.9)	26.2 (23.9, 28.5)
24	65.3	29.3 (26.7, 31.9)	28.8 (20.0, 37.7)

1 AGA, appropriate for gestational age; LAZ, length-for-age *z* score; MUAC, midupper arm circumference; ND, not determined; SGA, small for gestational age; WLZ, weight-for-length *z* score.

2 Missing data from 12 to 24 mo of follow-up were predominantly the result of administrative censorship because of economic constraints in June 2000.

3 Original data set.

4 Pooled across 50 imputed data sets.

5 In the Zimbabwe Vitamin A for Mothers and Babies trial, mother-infant pairs were randomly assigned ≤96 h after birth to 1 of 4 treatment groups as follows: maternal treatment [vitamin A supplementation (400,000 IU)], maternal placebo, infant treatment [vitamin A supplementation (50,000 IU)], and infant placebo. Full details of the trial have been published elsewhere (27, 28).

6 In multiparous mothers.

7 Detailed feeding information was collected from mothers at infant birth and 6 wk and 3 mo of age including whether any of 22 liquids (water, juice, tea, and cooking oil), milks (formula, fresh, and tinned), medicines (traditional, oral rehydration solution, and prescribed), or solid foods (porridge, sadza, fruit, vegetables, meat, and eggs) had been given to the infant. Breastfeeding was defined as exclusive, predominant, or mixed at 3 mo of age according to previously published definitions (31).

8 Total numbers of clinic visits or hospital admissions were collected from health facility records. The extent to which these data were missing from health-facility records could not be determined.

9 LAZ <−2.

We generated 50 complete data sets with the use of imputation and applied the KML algorithm to each as previously described. We followed the framework for multiple imputation in the cluster analysis proposed by Basagaña et al. (37) to select the final number of clusters (i.e., the number of clusters that was most-often selected as being optimal in the 50 complete data sets with the use of the Caliński-Harabasz index). The complete data sets for which the final number of clusters was chosen as optimal were retained for further analysis. We also used the Davies-Bouldin (38) and Ray-Turi (39) indexes to determine whether the optimal number of clusters that were chosen could be biased by the Caliński-Harabasz index. To graphically present the longitudinal growth in each cluster, we fitted generalized additive models of LAZ against infant age with the use of cubic splines with 7 knots for smoothing. Finally, we repeated these analyses with the use of data through 12 mo only to assess whether results were consistent when observational data were more complete.

### Multiple imputation of maternal height

Rates of missing data were very low for most baseline variables. However, maternal height (measured at the 6-wk visit) had a high frequency of missing values (Table 1). We opted to also impute missing maternal height for between-group comparisons and multinomial logistic regression analyses. We used predictive mean matching for this purpose with all available baseline variables included in the imputation model.

### Group comparison and multinomial regression

We used ANOVA and chi-square tests to determine whether sociodemographic and epidemiologic characteristics varied across trajectory groups. For variables with significant heterogeneity across groups, we performed pairwise tests for group differences with the use of generalized linear regression.

To determine sociodemographic and epidemiologic variables that explained the probability of trajectory-group membership, we used multivariable multinomial logistic regression. The outcome was defined as a nominal variable of LAZ trajectory-group membership and covariates were maternal education, age, midupper arm circumference, and height and infant sex, birth weight, length, and gestational age on the basis of the documented associations of these variables with linear infant growth (5, 8, 10, 13, 15, 40–45). To minimize the impact of collinearity on statistical inference, covariates were centered before model fitting. All results were pooled over the retained complete data sets with the use of the method proposed by Rubin (46). *P* values were corrected for multiple testing with the use of the Bonferroni method. All analyses were performed with R version 3.1.2 software with the use of kml (47), mice (35), and nnet (48) packages to implement the KML algorithm, multiple imputation via chained equations, and multinomial logistic regression, respectively.

## RESULTS

### Cohort description

Baseline characteristics for 3338 mother-infant pairs are shown in Table 1. Imputed data were very similar to the available, nonmissing data (Table 1, **Supplemental Figure 1**). Mothers had a mean age of 24.2 y (95% CI: 24.0, 24.3 y) at enrollment and a mean parity of 2.0 (95% CI: 1.9, 2.0). The majority of infants were born at term (mean: 39.3 gestational weeks; 95% CI: 39.3, 39.4 gestational weeks), and approximately one-half of the infants were boys (51.9%; 95% CI: 50.2%, 53.6%). Infant feeding at age 3 mo was predominantly mixed breastfeeding (43.4%; 95% CI: 41.8%, 45.1%); exclusive breastfeeding was infrequent (2.6%; 95% CI: 2.1%, 3.1%). One-fifth (19.0%) of infants were stunted (LAZ <−2) by age 12 mo, and almost one-third (29.3%) of infants were stunted by age 24 mo (Table 1). The mean ± SD length of follow-up of these 3338 mother-infant pairs was 21 ± 5 mo, and infants provided a mean 6 ± 2 visits at which the LAZ was measured.

### Clustering

**FIGURE 2:**
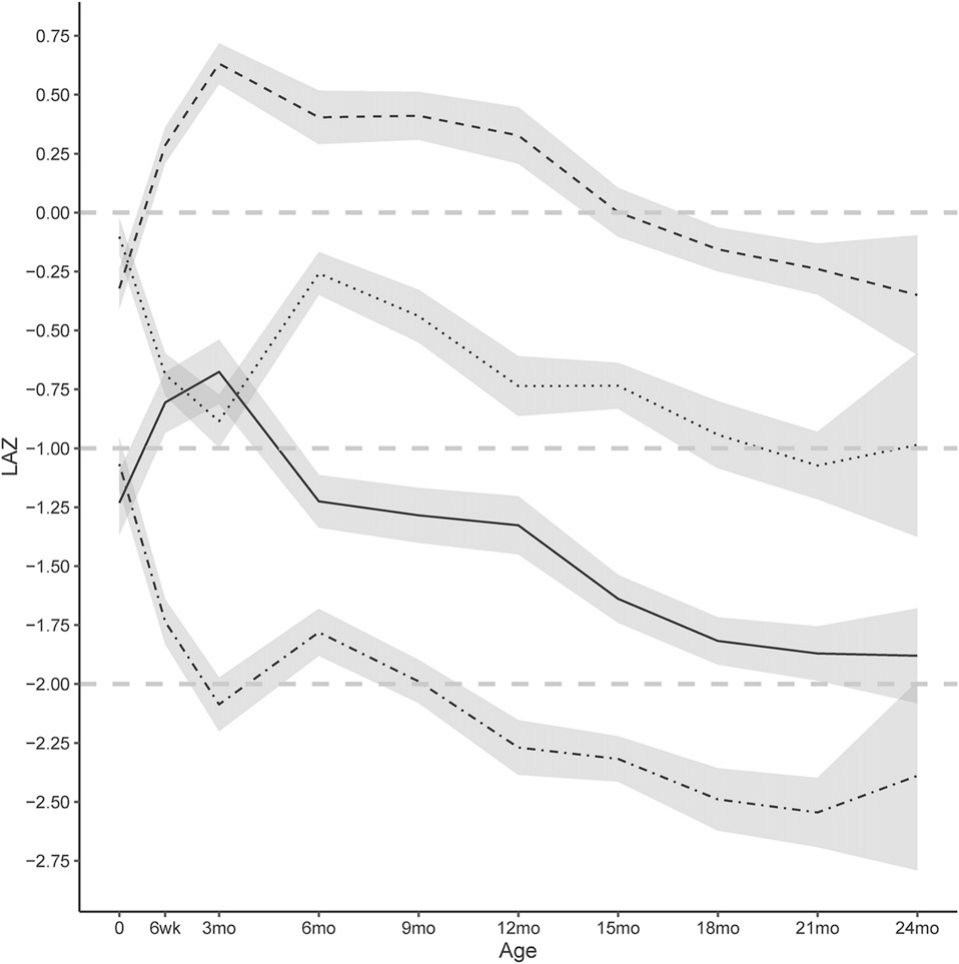
Mean (95% CI) linear growth trajectories from birth to 24 mo of age in 4 identified trajectory groups. *k*-Means clustering for longitudinal data was used to identify the optimal number of growth-trajectory groups of 9 possible groups. Four was chosen as the optimal number of groups. Trajectories were smoothed across 50 retained complete-data sets. The *k*-means method was designed to identify clusters even when they overlap because of both noise and crossing trajectories. Horizontal dashed lines indicate LAZ cutoffs for the WHO standard population median (LAZ: 0), mild stunting (LAZ <−1) and stunting (LAZ <−2). Groups are identified as follows: group A (dashed line), group B (dotted line), group C (solid line), and group D (dot-dashed line). Distributions of infants in each trajectory group were as follows: group A, 21.4% (19.4%, 23.4%); group B, 31.3% (28.9%, 33.8%); group C, 24.3% (22.2%, 26.4%); and group D, 23.0% (20.8%, 25.1%). LAZ, length-for-age *z* score.

KML identified 4 groups (hereafter termed groups A–D) as the optimal number of clusters in 50 complete data sets (100.0%) (**Supplemental Figure 2**) in which group A exhibited better growth and group D exhibited poorer growth. The 50 complete data sets were retained for further analyses. The optimal number of clusters chosen did not differ greatly with the cluster-quality index used. In 50 data sets, the proportions of infants identified as belonging to groups A–D were 21.4% (95% CI: 19.4%, 23.4%), 31.3% (95% CI: 28.9%, 33.8%), 24.3% (95% CI: 22.2%, 26.4%), and 23.0% (95% CI: 20.8%, 25.1%), respectively. The 4 groups were largely separated at birth when 5.4%, 2.5%, 24.3%, and 16.9% of infants were already stunted in the 4 respective groups. In all 4 groups, the LAZ declined between birth and 24 mo of age, but the rate and timing of decline varied between groups (**Figure 2**).

Infants in group A were persistently larger, on average, than other infants were and showed an initial increase in the LAZ until age 3 mo with a subsequent decline during the remainder of follow-up to a mean LAZ of −0.33 (95% CI: −0.59, −0.07) by 24 mo of age (**Table 2**, Figure 2). The prevalence of stunting in group A was low for the majority of follow-up with 2.4% (95% CI: 0.0%, 5.3%) of infants being stunted at 24 mo of age (Table 2). In group B, a linear decline in the mean LAZ was visible from 6 mo, and the prevalence of stunting was greater than in group A by 18 mo of age (5.5%; 95% CI: 2.9%, 8.2%). In group C, the mean LAZ was indicative of mild stunting at birth (−1.37; 95% CI: −1.51, −1.23). By 24 mo of age, 40.9% (95% CI: 30.9%, 50.9%) of infants in this group were stunted (Table 2). Group D contained infants with the greatest and earliest linear growth faltering. The mean LAZ in this group was −1.84 (95% CI: −1.94, −1.74) by age 6 mo, and 40.0% of these infants were stunted (95% CI: 35.4%, 44.6%). Infants in this group had a persistently lower LAZ than that of other groups throughout follow-up with a peak stunting prevalence of 70.8% (95% CI: 64.8%, 76.8%) at 18 mo of age (Table 2).

**TABLE 2:** Trajectory-group descriptions^1^

	Group, % of total cohort				
	A, 21.4 (19.4, 23.4)	B, 31.3 (28.9, 33.8)	C, 24.3 (22.25, 26.4)	D, 23.0 (20.8, 25.1)	*P*
Vitamin A treatment arm,^2^ %					
Maternal treatment–infant treatment	25.1 (21.6, 28.6)	24.7 (21.8, 27.7)	26.4 (22.9, 29.9)	24.2 (20.9, 27.5)	0.82
Maternal treatment–infant placebo	24.5 (21, 27.9)	24.5 (21.4, 27.5)	23.3 (20, 26.5)	25.6 (22.1, 29.1)	0.76
Maternal placebo–infant treatment	25.7 (22.2, 29.3)	24.9 (22.0, 27.8)	24.7 (21.4, 28.0)	24.2 (20.8, 27.6)	0.85
Maternal placebo–infant placebo	24.7 (21.2, 28.2)	25.9 (23.0, 28.7)	25.7 (22.2, 29.2)	26 (22.5, 29.5)	0.88
Maternal age, y	24.2 (23.8, 24.6)	24.2 (23.9, 24.6)	24.2 (23.8, 24.7)	24.0 (23.5, 24.4)	0.75
Maternal education, y	10.0 (9.9, 10.2)^C,D^	9.8 (9.7, 10.0)^C,D^	9.6 (9.4, 9.7)^A,B^	9.5 (9.3, 9.6)^A,B^	<0.01
Maternal height, cm	162.4 (161.7, 163.0)^B–D^	160.4 (159.9, 160.9)^A,C,D^	159.3 (158.7, 159.9)^A,B^	158.5 (158.0, 159.1)^A,B^	<0.01
Maternal MUAC, cm	26.3 (26.0, 26.6)^C,D^	26.1 (25.9, 26.3)^C,D^	25.6 (25.4, 25.9)^A,B^	25.7 (25.4, 25.9)^A,B^	<0.01
Cesarean delivery, %	7.6 (5.4, 9.8)	10.1 (8.2, 12.1)	6.5 (4.5, 8.5)	9.2 (6.9, 11.5)	0.07
Time since last birth,^3^ y	4.3 (4.1, 4.5)	4.4 (4.2, 4.6)	4.3 (4.1, 4.5)	4.3 (4.1, 4.5)	0.81
Children, *n*	1.9 (1.8, 2.0)	2, 0 (1.9, 2.1)	2.0 (1.9, 2.1)	2.0 (1.9, 2.2)	0.26
Parity, %					
Primiparous	50.4 (46.2, 54.5)	50.3 (47, 53.6)	48.6 (44.5, 52.7)	49.4 (45.4, 53.4)	0.88
Multiparous (2–3)	39.1 (35.0, 43.1)	36.6 (33.4, 39.9)	38.7 (34.8, 42.5)	35.9 (32.1, 39.7)	0.56
Multiparous (≥4)	10.6 (8.0, 13.1)	13.1 (10.9, 15.2)	12.7 (10.2, 15.3)	14.7 (12.0, 17.4)	0.20
Paternal education, y	10.8 (10.7, 10.9)^C,D^	10.7 (10.6, 10.8)	10.5 (10.4, 10.6)^A^	10.5 (10.4, 10.7)^A^	<0.01
Infant sex, M, %	45.6 (41.5, 49.7)^C,D^	48.7 (45.2, 52.1)^D^	53.9 (50.0, 57.7)^A^	60.1 (56.3, 63.8)^A,B^	<0.01
Gestational age, wk	39.2 (39.1, 39.3)^C,D^	39.1 (39.0, 39.2)^C,D^	38.8 (38.7, 38.97)^A,B^	38.7 (38.6, 38.8)^A,B^	<0.01
Term and AGA, %	94.3 (92.1, 96.5)^B–D^	88.2 (86.0, 90.4)^A,C,D^	81.1 (77.7, 84.5)^A,B,D^	70.4 (66.8, 73.9)^A–C^	<0.01
Preterm and AGA, %	2.7 (1.2, 4.3)^C,D^	7.2 (5.5, 9.0)	11.1 (8.5, 13.7)^A^	19.4 (16.5, 22.4)^A^	<0.01
Term and SGA, %	2.9 (1.5, 4.4)^B–D^	4.6 (3.2, 6.0)^A,D^	7.5 (5.4, 9.6)^A,D^	8.5 (6.3, 10.8)^A–C^	<0.01
Preterm and SGA, %	0.0 (0.0, 0.1)	0.1 (0.0, 0.2)^D^	0.3 (0.0, 0.8)	1.6 (0.7, 2.6)^B^	<0.01
Birth length, cm	48.8 (48.5, 49.0)^B–D^	49.6 (49.4, 49.8)^A,C,D^	47.0 (46.7, 47.3)^A,B,D^	48.0 (47.7, 48.2)^A–C^	<0.01
Birth weight, g	3204 (3169, 3239)^B–D^	3072 (3042, 3103)^A,C,D^	2918 (2877, 2958)^A,B,D^	2782 (2745, 2819)^A–C^	<0.01
Birth weight <2500 g, %	4.3 (2.6, 6.0)^C,D^	7.2 (5.4, 9.0)^C,D^	13.8 (10.8, 16.7)^A,B,D^	25.6 (22.1, 29.1)^A–C^	<0.01
Time until first breastfeeding, h	2.2 (1.9, 2.5)	2.8 (2.4, 3.2)	2.3 (2.0, 2.7)	2.7 (2.2, 3.2)	0.10
Breastfeeding,^4^ %					
None	39.9 (35.9, 43.9)	39.9 (36.2, 43.7)	37.4 (32.9, 42.0)	36.9 (32.9, 41.0)	0.49
Exclusive	2.6 (1.4, 3.9)	2.5 (1.5, 3.5)	2.9 (1.6, 4.1)	2.5 (1.3, 3.7)	0.96
Partial	15.4 (12.6, 18.2)	14.8 (12.4, 17.2)	15.1 (12.3, 17.9)	16.1 (13.3, 18.9)	0.89
Mixed	42.1 (38.1, 46.1)	42.8 (39.4, 46.1)	44.6 (40.4, 48.8)	44.4 (40.6, 48.3)	0.72
Clinic visits/infant by age in mo, *n*					
Birth to 6	1.21 (1.10, 1.33)	1.10 (1.01, 1.18)	1.14 (1.04, 1.25)	1.11 (1.00, 1.23)	0.42
6–12	0.79 (0.70, 0.88)	0.78 (0.71, 0.85)	0.83 (0.74, 0.92)	0.85 (0.76, 0.94)	0.55
12–18	0.61 (0.54, 0.68)	0.52 (0.47, 0.58)^C^	0.66 (0.59, 0.74)^B^	0.62 (0.54, 0.69)	0.03
18–24	0.34 (0.29, 0.39)	0.27 (0.23, 0.30)	0.35 (0.30, 0.40)	0.30 (0.25, 0.35)	0.07
Hospital admissions/infant by age in mo, *n*					
Birth to 6	0.05 (0.03, 0.07)	0.04 (0.02, 0.05)	0.05 (0.03, 0.07)	0.05 (0.03, 0.07)	0.69
6–12	0.01 (0.00, 0.02)	0.02 (0.01, 0.03)	0.02 (0.01, 0.03)	0.03 (0.02, 0.05)	0.05
12–18	0.01 (0.00, 0.02)	0.02 (0.01, 0.02)	0.02 (0.01, 0.03)	0.02 (0.01, 0.03)	0.75
18–24	0.02 (0.01, 0.02)	0.01 (0.00, 0.01)	0.01 (0.00, 0.02)	0.02 (0.01, 0.03)	0.34
WLZ, by age in mo					
Birth	0.08 (−0.04, 0.20)^B,D^	−0.81 (−0.91, −0.71)^A,C^	0.16 (0.04, 0.28)^B,D^	−0.86 (−1.00, −0.72)^A,C^	<0.01
6	0.72 (0.62, 0.82)^B–D^	0.39 (0.30, 0.48)^A^	0.43 (0.33, 0.53)^A^	0.22 (0.13, 0.32)^A^	<0.01
12	0.46 (0.36, 0.56)^B–D^	0.20 (0.12, 0.28)^A,C,D^	−0.10 (−0.18, −0.01)^A,B^	−0.24 (−0.32, −0.15)^A,B^	<0.01
18	0.41 (0.32, 0.50)^B–D^	0.14 (0.07, 0.22)^A,C,D^	−0.2 (−0.30, −0.10)^A,B^	−0.40 (−0.49, −0.31)^A,B^	<0.01
24	0.27 (0.17, 0.37)^C,D^	0.13 (0.04, 0.21)^D^	−0.07 (−0.17, 0.03)^A^	−0.27 (−0.37, −0.17)^A,B^	<0.01
LAZ, by age in mo					
Birth	−0.36 (−0.46, −0.27)^B–D^	0.01 (−0.08, 0.10)^A,C,D^	−1.37 (−1.51, −1.23)^A,B,D^	−0.92 (−1.04, −0.80)^A–C^	<0.01
6	0.37 (0.25, 0.48)^B–D^	−0.28 (−0.38, −0.18)^A,C,D^	−1.21 (−1.34, −1.09)^A,B,D^	−1.84 (−1.94, −1.74)^A–C^	<0.01
12	0.25 (0.09, 0.40)^B–D^	−0.67 (−0.85, −0.50)^A,C,D^	−1.42 (−1.57, −1.27)^A,B,D^	−2.23 (−2.39, −2.07)^A–C^	<0.01
18	−0.10 (−0.19, 0.00)^B–D^	−0.93 (−1.04, −0.82)^A,C,D^	−1.77 (−1.88, −1.66)^A,B,D^	−2.47 (−2.58, −2.36)^A–C^	<0.01
24	−0.33 (−0.59, −0.07)^B–D^	−0.99 (−1.39, −0.58)^A,C,D^	−1.86 (−2.07, −1.66)^A,B,D^	−2.39 (−2.80, −1.97)^A–C^	<0.01
Stunted,^5^ by age in mo, %					
Birth	5.4 (3.5, 7.4)^C,D^	2.5 (1.2, 3.8)^C,D^	24.3 (20.3, 28.4)^A,B^	16.9 (13.6, 20.2)^A,B^	<0.01
6	0.3 (0.0, 0.9)	1.3 (0.3, 2.3)^C,D^	16.3 (12.4, 20.2)^B,D^	40.0 (35.4, 44.6)^B,C^	<0.01
12	0.1 (0.0, 0.4)	2.6 (0.5, 4.7)^C,D^	19.2 (14.0, 24.5)^B,D^	59.2 (51.1, 67.3)^B,C^	<0.01
18	0.6 (0.0, 1.4)^B–D^	5.5 (2.9, 8.2)^A,C,D^	33.2 (27.6, 38.8)^A,B,D^	70.8 (64.8, 76.8)^A–C^	<0.01
24	2.4 (0.0, 5.3)^B–D^	9.8 (0.0, 19.7)^A,C,D^	40.9 (30.9, 50.9)^A,B,D^	66.7 (50.3, 83.2)^A–C^	<0.01

1 All values are means (95% CIs). *P* values were determined with the use of an ANOVA or χ^2^ analyses pooled across 50 retained complete data sets. ^A–D^When ANOVA or chi-square tests for heterogeneity were significant, pairwise group comparisons were performed with the use of linear or logistic regression pooled across 50 retained data sets. If the group presented in a column significantly differed from another group, a superscript letter is used to indicate the group it differed from. Pairwise significance was determined at α < 0.05 after Bonferroni correction. AGA, appropriate for gestational age; LAZ, length-for-age *z* score; MUAC, midupper arm circumference; SGA, small for gestational age; WLZ, weight-for-length *z* score.

2 In the Zimbabwe Vitamin A for Mothers and Babies trial, mother-infant pairs were randomly assigned ≤96 h after birth to 1 of 4 treatment groups as follows: maternal treatment [vitamin A supplementation (400,000 IU)], maternal placebo, infant treatment [vitamin A supplementation (50,000 IU)], and infant placebo. Full details of the trial have been published elsewhere (27, 28).

3 In multiparous mothers.

4 Detailed feeding information was collected from mothers at infant birth and 6 wk and 3 mo of age including whether any of 22 liquids (water, juice, tea, and cooking oil), milks (formula, fresh, and tinned), medicines (traditional, oral rehydration solution, and prescribed), or solid foods (porridge, sadza, fruit, vegetables, meat, and eggs) had been given to the infant. Breastfeeding was defined as exclusive, predominant, or mixed at 3 mo of age according to previously published definitions (31).

5 LAZ <−2.

A comparison of observed and imputed mean trajectories in each group illustrated that the imputation of unobserved values did not create subgroups with different growth curves. We would have expected *1*) that observed and imputed mean values would have differed within each group and *2*) that overall group means would have more-closely tracked the imputed values than the observed values if the imputations were driving the group trajectories (**Supplemental Figure 3**).

An analysis of data that was restricted to the first 12 mo of life also identified 4 clusters as being optimal (**Supplemental Figure 4**). The mean trajectories in these groups (**Supplemental Figure 5**) corresponded well with the patterns that were identified with the use of 24 mo of data, and there was a substantial overlap in the assignment of infants to equivalent 12-mo and 24-mo groups (**Supplemental Table 1**).

### Comparison of group characteristics

Group A infants were most different in their baseline characteristics from those of other infants (Table 2). Infants in group A (best growth) had greater maternal education (10.0 y), maternal midupper arm circumference (26.3 cm), maternal height (162.4 cm), infant birth length (48.8 cm), and infant birth weight (3204 g) compared with the measures of other infants. More group A infants were also born appropriate for gestational age (AGA) at term (94.3%), and fewer infants in group A were term SGA (2.9%) (Table 2). Group D (worst growth) had infants with significantly lower birth weight (2782 g), more term SGA (8.5%), and less term AGA (70.4%) than in the other groups. Overall, across groups A–D, maternal height, birth weight, proportion of infants who were term AGA, and the LAZ at each 6-mo visit after birth progressively declined, whereas the proportion of infants who were boys, were term SGA, or had LBW progressively increased.

### Multinomial regression

To investigate factors that distinguished group membership, we report our multiple regression model with the use of group C as the referent in **Table 3**. Baseline maternal education and height and infant sex, birth length, and birth weight were significantly associated with the group membership. Each 1-y increase in maternal education [adjusted OR (aOR): 1.13; 95% CI: 1.06, 1.22], 1-cm increase in maternal height (aOR: 1.08; 95% CI: 1.05, 1.10), 1-cm increase in infant birth length (aOR: 1.25; 95% CI: 1.14, 1.38), and 100-g increase in infant birth weight (aOR: 1.13; 95% CI: 1.09, 1.18) was associated with increased odds of membership in group A. Male infant sex was associated with reduced odds of membership in group A (aOR: 0.50; 95% CI: 0.38, 0.65). Infant birth length was also associated with increased odds of membership in group B (aOR: 1.76; 95% CI: 1.59,1.94), whereas infant sex was associated with reduced odds of membership in group B (aOR: 0.58; 95% CI: 0.45, 0.75), and infant birth weight was associated with reduced odds of membership in group D (aOR: 0.85; 95% CI: 0.82, 0.88). The probability of group membership was not determined by which group was chosen as the referent, and thus, changing the referent group did not change the results of our model (**Supplemental Table 2**). Finally, to investigate whether the 4 groups that were identified could be explained by the season of birth, we generated box plots of birth weight by the month of birth for each group (**Supplemental Figure 6**). The decline in birth weight from groups A to D was consistent across the month of birth, thereby indicating that the season of birth did not explain the clusters.

**TABLE 3:** Full multinomial regression model of trajectory-group membership and maternal and infant characteristics at baseline^1^

	Trajectory group,^2^ %		
	A, 21.4 (19.4, 23.4)	B, 31.3 (28.9, 33.8)	D, 23.0 (20.8, 25.1)
Maternal age, y	0.97 (0.95, 1.00)	0.98 (0.96, 1.01)	0.98 (0.96, 1.01)
Maternal education, y	1.13 (1.06, 1.22)^3^	1.07 (1.00, 1.14)	0.97 (0.91, 1.03)
Maternal height, cm	1.08 (1.05, 1.10)^3^	1.03 (1.01, 1.05)^4^	0.98 (0.96, 1.00)
Maternal MUAC,^5^ cm	1.06 (1.01, 1.11)	1.05 (1.01, 1.10)	1.04 (1.00, 1.09)
Infant sex, M compared with F	0.50 (0.38, 0.65)^3^	0.58 (0.45, 0.75)^3^	1.33 (1.04, 1.68)
Gestational age, wk	0.99 (0.90, 1.09)	0.99 (0.90, 1.09)	1.01 (0.93, 1.11)
Birth length, cm	1.25 (1.14, 1.37)^3^	1.76 (1.59, 1.94)^3^	1.32 (1.20, 1.44)^3^
Birth weight, g	1.13 (1.09, 1.18)^3^	0.96 (0.92, 1.00)	0.85 (0.82, 0.88)^3^

1 Unless otherwise stated, all values are ORs (95% CIs). All ORs are per 1-unit increase in exposure except for birth weight, for which adjusted ORs are per 100-g change in birth weight.

2 A multinomial logistic regression model with the covariates reported in the table was fit with group C as the referent.

3 Bonferroni corrected *P* < 0.01.

4 Bonferroni-corrected *P* < 0.05.

5 MUAC, midupper arm circumference.

## DISCUSSION

In this longitudinal birth cohort of HIV-unexposed mother-infant pairs from Zimbabwe, the mean LAZ was suboptimal at birth and subsequently declined between birth and 24 mo of age. This pattern of linear growth failure was consistent with survey data from countries in Africa and Asia (17). However, we identified 4 linear growth–trajectory groups that differed in the timing and magnitude of growth decline. The probability of group membership was predicted by the following 5 factors: maternal height and education and infant sex, birth length, and birth weight.

To our knowledge, only one other publication has grouped infants according to their individual linear growth trajectories (49). The study, which was from a developed country (Portugal), identified 4 trajectory groups. Compared with the group with the best growth, the group with the worst growth were shorter at birth (mean: 45.4 compared with 49.7 cm; *P* = 0.003) and weighed less (mean: 2.59 compared with 3.49 kg; *P* < 0.001). The study did not report maternal determinants of growth.

Although we did not investigate stunting (LAZ <−2) as an outcome, our findings were consistent with the literature on risk factors for stunting. Analyses of multicountry data have shown 2.90-times greater odds (95% CI: 2.56, 3.33) of stunting in 12- to 60-mo-old children who were born with LBW than in children with normal birth weight (5). Multicountry analyses have also shown a reduction in risk of stunting (RR: 0.968; 95% CI: 0.967, 0.968) (41) and gains in height-for-age *z* scores (β: 0.037; 95% CI: 0.033, 0.040) (40) with increasing maternal height. Associations between stunting and birth weight (15, 44) or maternal height (3, 50) have also been identified in other studies. Disparities in linear infant growth can also be explained by maternal education (13, 43, 51). Finally, sex differences in stunting have been well described: 35.7% of boys and 28.3% of girls <5 y of age in Zimbabwe were stunted in 2010–2011 (52), which are typical of findings from several countries particularly in sub-Saharan Africa (53, 54). We have extended these findings by showing that these same variables also explain membership in different growth-trajectory groups.

Birth weight and length reflect the effect of the intrauterine environment on fetal growth (26) and the overall adequacy of nutrient intake during pregnancy to meet maternal and fetal needs (45). Maternal nutrition may also produce epigenetic influences in offspring (55–59), which has been suggested to affect infant growth (60). Short maternal stature poses constraints on fetal growth (40, 41) and reflects the combined effect of genetics, nutrition, and environment during the mother’s own growth and development, which may impair a mother’s capacity to deliver nutrients to her fetus when she reaches reproductive age (41). Although the maternal educational level is very commonly associated with stunting, the causal mechanism is not known; this variable may be a proxy for socioeconomic status or a determinant of the maternal-infant interaction, use of health services, or maternal diet. Finally, boys have shown higher rates of clinical illness in early life (61–63), potentially because of differences in inflammatory and immune responses compared with girls, as has been suggested by sex differences in the immune response to vaccination (64) and infectious diseases (63). It has also been suggested that natural selection may favor maximizing reproductive fitness, thereby resulting in higher morbidity and mortality rates in male infants to compensate for greater male birth rates (65, 66).

Overall, these results suggest that transgenerational effects are an important determinant of growth trajectories, and interventions targeting pregnant mothers as well as girls entering their reproductive years are required. Nutritional supplementation of girls aged 7–15 y (67) and during the first 3 y of life (68) produced significant improvements in birth weights, postnatal heights, and weights of their offspring. Micronutrient and macronutrient supplementation during pregnancy also improved birth sizes (25) and may be important determinants of infant growth (25, 69, 70). A greater focus on maternal nutrition (71), stunted families (72), and socioeconomic factors (including female education, improved access to healthcare, and improved water, sanitation, and hygiene) (51, 73) are likely requirements of normal infant growth.

However, our finding that the mean LAZ sharply declined between birth and 24 mo of age in all clusters reaffirmed the importance of postnatal interventions. Even mild stunting (LAZ ≥−2 but <−1) is associated with increased risk of mortality from infections (74). Thus, postnatal interventions that directly address the diets of infants and young children (25) as well as nutrition-sensitive interventions that address access to food and nutrient use (e.g., agriculture, social safety net, early child development, and schooling (75) as well as sanitation and hygiene (76)] will be critical to the prevention of stunting.

The application of multiple imputations allowed us to retain all available subjects in our analyses and to avoid potential bias from the exclusion of infants without complete data through 24 mo of follow-up. The imputed data were very similar to the observed data at each visit. The determination of the correct number of clusters is fundamental to a cluster analysis, but there is currently no optimal method to validate the choice of a final cluster number (32). We used the Caliński-Harabasz criterion because it has been shown to outperform other common measures of cluster quality on simulated data (77, 78) and is robust to a number of factors that may affect cluster-number selection (79). The criterion also performs well in the identification of longitudinal trajectory clusters even when clusters overlap and their trajectories cross (32). Discrepancies between the Davies-Bouldin, Ray-Turi, and Caliński-Harabasz indexes regarding the best number of clusters were not large, and 2 of the 3 indexes identified 4 clusters as being optimal. Our results were also robust to the restriction of a cluster analysis to the first 12 mo of life when more complete observed data were available. In addition, the use of multiple imputations allowed for some of the uncertainty in cluster-membership assignment to be accounted for in the analyses (37) because individuals were not assigned to a cluster with 100% certainty. The trajectory groups that we identified may not be generalizable to infants outside of this cohort. Several key variables, such as paternal height, adequacy of infant dietary intake, and household access to clean water and sanitation, were not available for us to assess as predictors of trajectory membership. We could not assess whether longitudinal, time-varying exposures could have further explained the group membership with the use of multinomial logistic regression. Analyses of birth cohorts in other developing countries are needed to confirm these results.

In conclusion, we performed our analyses in a large, HIV-unexposed, urban birth cohort from Zimbabwe who were followed from birth through 2 y of age and identified 4 patterns of postnatal linear growth. The following 5 antenatal factors explained the probability of infant membership in any given group: maternal height and education and infant birth length, birth weight, and sex. These results show the importance of focusing on multisectoral interventions to improve the education, health, and nutritional status of females before pregnancy to improve transgenerational effects on infant growth. However, the observation that LAZ trajectories sharply decline between birth and 24 mo of age, even in the group with the best antenatal factors, shows the concomitant importance of postnatal stunting-prevention interventions.

## Footnotes

9 Abbreviations used: AGA, appropriate for gestational age; aOR, adjusted OR; KML, *k*-means clustering for longitudinal data; LAZ, length-for-age *z* score; LBW, low birth weight; SGA, small for gestational age.
